# Studying the effect of self-selected background music on reading task with eye movements

**DOI:** 10.1038/s41598-023-28426-1

**Published:** 2023-01-30

**Authors:** Ying Que, Yueyuan Zheng, Janet H. Hsiao, Xiao Hu

**Affiliations:** 1 Faculty of Education, The University of Hong Kong, Hong Kong SAR, China; 2 Department of Psychology, The University of Hong Kong, Hong Kong SAR, China; 3 The State Key Laboratory of Brain and Cognitive Sciences, The University of Hong Kong, Hong Kong SAR, China; 4 The Institute of Data Science, The University of Hong Kong, Hong Kong SAR, China; 5 Shenzhen Institute of Research and Innovation, The University of Hong Kong, Shenzhen, China

**Keywords:** Psychology, Human behaviour

## Abstract

Using background music (BGM) during learning is a common behavior, yet whether BGM can facilitate or hinder learning remains inconclusive and the underlying mechanism is largely an open question. This study aims to elucidate the effect of self-selected BGM on reading task for learners with different characteristics. Particularly, learners’ reading task performance, metacognition, and eye movements were examined, in relation to their personal traits including language proficiency, working memory capacity, music experience and personality. Data were collected from a between-subject experiment with 100 non-native English speakers who were randomly assigned into two groups. Those in the experimental group read English passages with music of their own choice played in the background, while those in the control group performed the same task in silence. Results showed no salient differences on passage comprehension accuracy or metacognition between the two groups. Comparisons on fine-grained eye movement measures reveal that BGM imposed heavier cognitive load on post-lexical processes but not on lexical processes. It was also revealed that students with higher English proficiency level or more frequent BGM usage in daily self-learning/reading experienced less cognitive load when reading with their BGM, whereas students with higher working memory capacity (WMC) invested more mental effort than those with lower WMC in the BGM condition. These findings further scientific understanding of how BGM interacts with cognitive tasks in the foreground, and provide practical guidance for learners and learning environment designers on making the most of BGM for instruction and learning.

## Introduction

Music is extensively used in everyday contexts, for special occasions, entertainment, instructional activities, or accompanying working or studying in the background^1^. Studies in different domains, including education^2,3^, psychology^4,5^, and information science^1^, have all demonstrated that many learners (sometimes over half of the samples) in all levels from primary to graduate schools^1^ have the habit of studying with background music (BGM), and some of them had music playing in the background extensively^3^, even 90% of the time^2^. Despite the popularity of the behavior, previous studies have yielded mixed results when it comes to the effect of BGM on cognitive tasks performance^3^. Some researchers reported benefits, such as enhancing mood or heightening arousal^4^, which in turn, might indirectly affect learning, while others found detrimental effects, such as cognitive overload, cognitive dissonance, or distraction^5,6^.

The reasons for different findings are complex, as each study differs in BGM stimuli, methodological features, nature of the cognitive tasks, and learner characteristics^3,7^. A systematic review of literature on BGM and learning from 2008 to 2018^3^ suggested that the BGM in most existing studies was provided by the researchers in which participating students listened to the same pieces when they conducted cognitive tasks in the forefront. This may raise issues on ecological validity as students’ musical options were limited. In other words, not all participating students would listen to BGM selected by the researchers in their daily life. This study thus aims to fill the gap by using student provided BGM, namely music they would use as background for learning in their daily life. In contrast to most existing studies that used a small number of music pieces^3–5^, this paper makes a methodological contribution by broadening the music selection. Besides, in previous research, learner characteristics such as working memory capacity (WMC)^5^, degree of extraversion^8^, and music listening habits/experience^3^ were identified as relevant. Furthermore, language proficiency was found critical for reading comprehension, especially for non-native speakers^9^. However, few empirical studies have examined all these learner characteristics simultaneously and thus could not assess which factors were more important than others. Therefore, this study also attempts to fill this gap by analyzing these individual factors at the same time.

Methodologically, while previous studies on reading and BGM were mostly based on self-report measures and reading tests^3–5,10–12^, the development of eye-tracking technology provides a more objective approach with fine-grained eye movement data^13,14^. So far few studies have employed eye-tracking technology to probe how BGM impacts the reading process^10,15,16^, and even fewer delved into finer eye-movement measures such as those in lexical and post-lexical levels^16,17^. Besides reading task performance which aimed to assess how well students accomplished a reading task and included measures of passage comprehension accuracy and passage reading time, this study also examined self-reported metacognition and eye movement measures to answer the following two research questions (RQs):

- *RQ1* Is there any difference in reading task performance, self-reported metacognition, or eye movement patterns between students who read with and without BGM?
- *RQ2* To what extent are learners’ individual factors (e.g., English proficiency, WMC, extraversion, BGM listening habits, music expertise) related to reading task performance, self-reported metacognition, or eye movement patterns in conditions with and without BGM?

To answer these questions, we conducted an experiment with 100 English as a Second Language (ESL) learners who were randomly divided into experimental and control groups. The former listened to self-provided music during English reading tasks while the latter performed the same reading tasks in silence. Findings of this study provide more evidence on the effect of self-selected BGM on reading, which is more ecologically valid than those in most previous studies employing music selected by researchers. On the theoretical side, this study can further the scientific understanding of how BGM interacts with cognitive tasks in the foreground, particularly on complex cognition and oculomotor behaviors in reading tasks. On the methodological side, since reading comprehension is a cognitive process and eye movements can be used to explain/predict perceptual attention^18^, information acquisition processes^19^ and information integration processes^16^, this study employed fine-grained eye movement measures to understand how students read. Fine-grained measures are advantageous in revealing different levels of cognition (e.g., first-pass fixation duration can reflect early lexical processing; regressive eye movements can reflect difficulties in post-lexical semantic integration)^13,20^. On the practical side, this study can provide implications for learners and those who design learning environments for learners in optimizing reading and self-learning through making personalized decisions on BGM usage.

## Literature review

### Theoretical explanations for the effects of background music

As summarized by a recent systematic review on the effects of BGM on learning^3^, findings on this topic are inconsistent, including positive, negative or no effect. From the cognitive perspective, the mixed results can be explained by Cognitive Load Theory (CLT) introduced by Sweller et al.^21^. CLT suggests that working memory capacity (WMC) is finite and cognitive load is a basic measurement of how much “space” in WMC is currently being consumed^21^. If the experienced load of a task exceeds a learner’s capacity, task performance would be stifled^22^. Extraneous load is a form of cognitive load, which is exerted by the way information is presented (e.g., visual, audio, text) and is deemed as unproductive load in that it does not directly contribute to construction of schemas (i.e., knowledge structures organized around core concepts)^21,23,24^. BGM may thus be regarded as a type of extraneous load to explain BGM’s negative impacts^25^ when the BGM is not strategically integrated to the instruction or the learning task as a part of learning design^26^.

However, it may be the case that a low level of extraneous load induced by BGM could motivate learners to devote more time into a learning task, thus potentially leading to positive effects on reading over the longer term^25^. According to the arousal-mood-hypothesis (AMH)^27^, music influences learning by affecting the learner’s arousal and mood^28^ which in turn are associated with the accomplishment of learning activities^29^. Furthermore, allowing learners to exercise an autonomous decision regarding the selection of their preferred BGM can enhance their intrinsic motivation^30^. These two theories (CLT and AMH) were complementary rather than contradictory. Both served as guidelines for attaining the goal of employing the BGM to help learners maintain good mood, motivation, engagement, but not hindered the learning tasks in the forefront.

### Measuring the effect of background music with eye movement

Eye-tracking provides a useful method to record every moment of eye movement during reading, based on which the cognitive process of the reader can be inferred^14^. Successful reading comprehension involves two stages of information processing. The early stage is to recognize individual words in the text (i.e., lexical access). The late stage is to connect individual words and reconstruct content, also known as post-lexical processes^16,31^. Eye movement can indicate cognitive load imposed on readers at both early (lexical access) and late (post-lexical process) stages of information processing^16,17^. The early processing is typically represented by first pass measures such as first pass fixation duration and first pass saccade amplitude where “first pass” refers to the first time a reader looks at a region (i.e., word) as opposed to re-reading^13^. The late processing can be evaluated by measures of rereading the text or regressions to earlier portions of the text^13^. Computational models such as the E-Z Reader proposed by Reichle et al.^20^ found that difficulties from post-lexical processes (e.g., semantic integration) would result in more fixations and/or regressive eye movements. Increased regressive eye movements were also found to be connected to in-depth text processing^32,33^.

Notwithstanding the fact that eye-tracking has been employed for studying reading comprehension and providing effective indicators of learners’ cognitive processing, few studies^15,16^ investigated readers’ eye movements in the presence of their self-selected BGM. At the lexical access stage, eye movement is associated with the difficulty of word processing. For skilled readers, certain reading behavior such as the identification of most high-frequency words can be automatic^34^, in which they may demonstrate a well-developed visual routine^35^, and thereby the influence of BGM on processing of such words may not be salient^16^. At the post-lexical stage, to compensate for the possible distraction caused by BGM, readers’ eye movement may involve more regressions and longer viewing time^16^. As extant literature suggested, eye movement strategy (e.g., successful and effective regressive movements) could demonstrate readers’ abilities in controlling the ongoing process of reading comprehension in silence^36^ and with self-selected BGM^16^. However, which traits of the readers might be related to the eye movement patterns when reading with their self-selected BGM has not been clarified.

### Interactions among individual traits, reading task, and background music

Individual differences appear in almost all studies on BGM and learning. Working memory capacity (WMC) is a factor that can moderate the effect of BGM on reading comprehension^5,37^. Baddeley and Hitch^38^ proposed that working memory (WM) represents a control system with limits in processing and storage capacity. More recent research^5,37^ showed that individuals with high WMC performed better with BGM in reading-comprehension tasks than those with low WMC. It can result from limited capacity of human working memory. Since information from different channels of WM (i.e., visual and verbal/auditory WM) can compete for limited WM resources, WM capacity could be overloaded owing to the presence of BGM while reading^5,39^. Additionally, the nature of BGM (e.g., lyrics, tempo, genre, volume) can indirectly influence learning^7,26^. While these features of music might overload auditory channel, with careful manipulation, they might also reduce cognitive load and increase attention.

Personality traits such as extraversion have been considered when discussing the role that BGM played in cognitive tasks^15,40^. Based on Eysenck’s theory of personality^41^, some scholars hypothesized that extraverts may benefit from reading with music, while introverts may not as they are more sensitive to arousal stimuli including music. However, according to a review^8^ that examined extraversion as a moderator in how BGM affects cognitive task performance, there was as much evidence in favor of Eysenck’s theory as there was against it.

BGM listening habits and preference are other factors worthy of consideration. Both positive and negative connections between these factors and study performances were reported. An experimental study conducted by Etaugh and Ptasnik^42^ showed that participants who were accustomed to listening to music while studying performed better in verbal learning tasks than those who were not habitually having music played in the background. However, Anderson and Fuller^11^ found that students who stated they enjoyed listening to BGM while studying in daily life scored worse on text comprehension tests in both music and non-music environments than those who did not enjoy BGM in general.

Besides, learners’ musical expertise was also identified as an important factor^7^. Nonetheless, a recent systematic review^3^ presents that a large body of research on the effects of BGM on learning did not report learners’ musical expertise or their training on music. It is noted that few existing studies on BGM and learning have inspected or compared all the aforementioned personal factors at the same time, and this study aims to bridge the research gap.

## Methods

Due to its advantages in controlling contextual variables and providing clear evidence for or against pre-specified hypotheses^43^, a laboratory-based experimental research design was adopted to answer the research questions raised above, with details explained in the following sub-sections. In particular, we adopted a between-subjects experiment design which can minimize the learning and transfer across conditions. Half of the participants were assigned to the experimental condition (BGM group) where they listened to BGM during the reading task, while the other half to the control condition (Silence group) where they performed the reading task in silence. This study was approved by the Human Research Ethics Committee (HREC) of the University of Hong Kong (HREC reference number: EA1802092). All methods were performed in accordance with the American Psychological Association ethical standards.

### Participants

The participants were recruited from English as Second Language (ESL) learners from a major comprehensive university. Participants should be between 18 and 35 years old and have no visual, hearing, or learning impairment.

### Reading materials

In this study, participants were tasked to read nine short English passages. The passages were selected from online exercises available at: https://gre.koolearn.com/yuedu/, http://gre.kmf.com/practise/rc for preparing Graduate Record Examinations (GRE), and then validated by three experts in the field (English teachers in an English-medium university). An index of passage difficulty level, Flesch-Kincaid grade^44^ was calculated using readable.io for each passage. As shown in Table 1, the nine passages were evenly distributed across three difficulty levels based on the F-K grade: Easy (mean grade 13.2), medium (17.4), and hard (21.0). The lengths of the passages were also comparable, with an average of 219, 217, and 217 words for passages in the easy, medium, and hard levels respectively.

**Table 1:** Information of the passages.

Passage no	Passage difficulty level	Theme	Flesch-Kincaid grade	Word count
P1	Easy	Astronomy	12.9	210
P2	Easy	Astrobiology	13.6	239
P3	Easy	Physics	13.3	210
P4	Medium	Archaeology	17.0	210
P5	Medium	History	17.3	227
P6	Medium	Literature	17.9	214
P7	Hard	Biology	20.6	221
P8	Hard	Sociology	20.8	212
P9	Hard	Anthropology	21.5	218

The passages covered different academic disciplines (i.e., astronomy, astrobiology, physics, etc.), for counterbalancing possible bias caused by participants’ previous knowledge. To assess passage comprehension accuracy, we designed two multiple choice questions (MCQs) for each passage, one text-based and one inferential^45^. The former was conceptually simple and only required shallow understanding of the content (e.g., “According to the passage, which of the following statement is true of Mars?”), while the latter required reasoning and deep understanding of the passage’s content (e.g., “Which of the following is the primary purpose of the passage?”). According to Bloom’s Taxonomy of learning objectives^46^, 50% (N = 9) of the MCQs targeted factual knowledge, 39% (N = 7) targeted metacognitive knowledge, and 11% (N = 2) targeted conceptual knowledge. For each question, four response alternatives were presented, including a target (the one that correctly answered the question), a near-miss (the one that sounded almost correct but not), a thematic miss (the one that was relevant to the theme of the content but irrelevant to the question) and a miss (the one that was totally irrelevant).

### Music stimuli

To achieve a higher level of ecological validity, the music stimuli utilized in this study were self-provided by the participants. Before the participants came to the experiment, we asked them to provide music audio files or playlists with a total duration of 40 or more minutes. These music tracks should be what the participants often liked to listen to during self-learning or reading in their daily lives. For participants who did not use to listen to music while studying, they were instructed to provide music they would not mind listening to while reading. Researchers pre-processed and standardized the music by adding fading in and fading out effects and shortening silent segments if any. The headphone was adjusted to a range from 65 to 75 dB(A), which was acceptable for learning as suggested in the literature^12^.

### Experiment procedure

**Figure 1:**
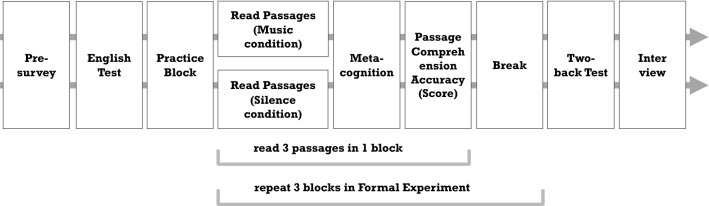
Procedure of the experiment.

Before the experiment started, participants signed an informed consent form which detailed the experiment procedure, data privacy protection measures, and participants’ rights. As shown in Fig. 1, the experiment started with a pre-questionnaire on participants’ demographic information (e.g., age, gender), English learning experience, personality traits, training on music, and BGM listening habits during reading or self-learning. Based on the information, we balanced the BGM and Silence conditions so that participants in the two groups were largely comparable. Participants were also asked to take the online LexTale test at http://www.lextale.com/takethetest.html to gauge their general English proficiency.

The facilitator then guided each participant to become familiar with the experiment procedure through a practice block that included a sample English text (different from the nine passages used in the formal experiment) and BGM which was selected by the authors and consistent across participants. It should be noted no data were collected from the practice block. After practice, the formal experiment began. It consisted of three blocks, each of which contained three English passages with one in each difficulty level (c.f., Table 1). The order of the three blocks and that of the three passages in a block were randomized using a Latin square design across participants for counterbalance.

Right before participants started a reading block, the eye tracker was calibrated to track the movement of participants’ dominant eyes. Before they started each passage, drift correction with the eye-tracker was performed to ensure the eye-tracking data were accurate. During reading, the BGM group read with participants’ self-provided music playing in the background, while for the Silence group, the background was in silence. All participants were asked to memorize as much of the passage’s content as possible, since they were not allowed to return to the passage once they entered the question answering stage. Upon completing a passage, three metacognitive questions on engagement, difficulty, and understanding were presented in sequence, followed by two MCQs (one text-based; the other inferential) testing passage comprehension accuracy. As the metacognition questions were about passage reading, they were presented right after the passages to avoid possible confusion of the participants. To ensure the question answering conditions in the two groups were comparable, music was paused for the BGM group during answering the questions. There was no time constraint when participants read or answered questions. After submitting answers to the MCQs, the next passage started. Meanwhile, the music resumed for the BGM group. Upon completing a block, participants were given a 2 min break to allay possible tiredness.

After completing all three blocks of reading tasks, participants took a two-back test which aimed to measure their working memory capacity. An exit-interview was then conducted to elicit their thoughts and feelings on studying with BGM and experiences in the experiment, with example questions asking “How do you like studying with music in the background? Why or why not?” and “What do you think about this experiment?”. It is noteworthy that the participants did not know whether they were in BGM or Silence condition until the experiment was completed. The experiment lasted 100-120 minutes in total. At the end of the experiment, each participant was paid a nominal remuneration of about 20 US dollars.

### Apparatus

EyeLink 1000 plus (Tower mount model; SR research) was utilized to record eye movements with a sampling rate of 2k Hz during reading. A chin and forehead rest were used to minimize participants’ head movements. The resolution of the monitor is 1280 × 1024 pixels. A viewing distance was set as around 56 cm and each English letter extended about 0.3 degrees of horizontal visual angle to simulate natural reading situations^47^. Default settings for cognitive research were used for the eye tracker, including saccade motion threshold set as 0.1° of visual angle; saccade velocity threshold set as 30°/s; saccade acceleration threshold set as 8000°/s^2^; and saccade pursuit fixup set as 60°/s^70^. For calibration, a nine-point calibration/validation procedure was conducted at the beginning of each block. In addition, at the beginning of each passage, drift correction was conducted to detect the difference between the current fixation position and the one based on last calibration. If the difference exceeded 1° of visual angle, re-calibration was performed.

### Reading outcome measures

The following reading outcome measures at behavioral, self-reported, and eye-tracking levels were applied to answer the research questions raised in this study.

*Reading Task Performance* can be measured by the following two indicators^48^.

- Passage Comprehension Accuracy (score) is represented by the proportion of correctly answered post-reading comprehension MCQs (including text-based and inference questions) among all questions.
- Passage Reading Time refers to the time difference between the time point when a passage started to be presented on the screen and the time point when participants clicked the “continue” button to proceed to the question answering stage.

*Metacognition* was assessed by three subjective ratings: engagement extent in reading, passage difficulty degree, and understanding level. The items were adapted from previous literature^49–51^ and designed on five-point Likert scales, with 1 indicating the lowest rating (i.e., not engaged at all, not difficult at all, not understand at all) and 5 indicating the highest rating (i.e., very engaged, very difficult, completely understand).

- Engagement Extent is evaluated by the question “How engaged were you during reading?”.
- Difficulty Degree is assessed by the question “Do you think this passage is difficult?”.
- Understanding Level is determined by the question “To what extent do you understand this passage?”.

*Eye-tracking measures* of each participant during passage reading were captured in six classical constructs: fixation, saccade, regression, word skipping, pupillary response, and blinking.

**Figure 2:**
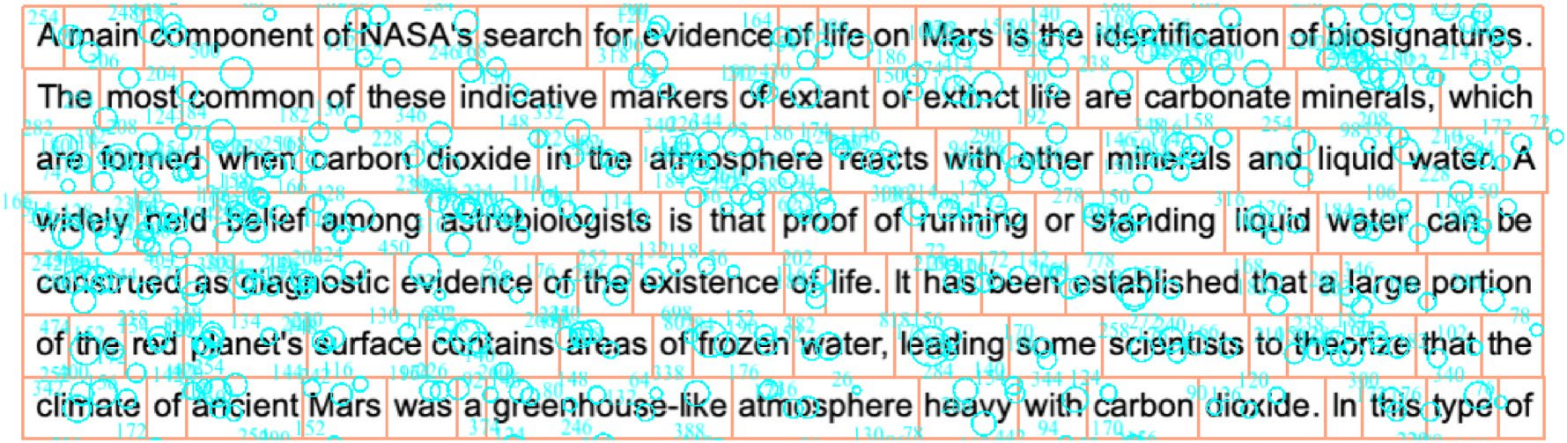
An example fixation graph of one single participant with areas of interest segmented by word. *Note.* The size of fixation circle is proportional to the fixation duration.

- Fixation is the most common type of eye movement events which occur when the eye stays stationary for an extended length of time^52^. In the case of English reading, a fixation usually lasts around 200–300 ms (ms) on a word^53^. As a marker of cognitive load, more and longer fixations often imply the ongoing process is cognitively demanding^54^. The current study adopted four fixation-related measures: (1) First Fixation Duration and (2) First Pass Dwell Time are local word-level fixation measures; (3) Total Fixation Count and (4) Total Fixation Duration are global passage-level fixation measures.
- Saccade is a kind of rapid eye movements between two fixations at a speed of up to 500 degrees per second, which can reflect reading efficiency and text processing difficulty^53^. For example, shorter saccades were found to be associated with readers with poor reading abilities^55^ or texts with high difficulty^32^. Saccade amplitude refers to the distance travelled by the eyes between two fixations and is measured in the unit of degrees of visual angle. In this study, one measure related to saccade amplitude is included: First Pass Saccade Amplitude is a local word-level forward saccade measure.
- Regression refers to eye movement from the currently fixated word back to one of the words encountered before. In general, a reading task with higher mental workload would result in more regressions^15,32^. In this study, Total Regression Count, a global passage-level backward saccade measure, was adopted and calculated by a method provided by EyeLink DataViewer. The first step of the method was to automatically segment words in a passage into individual interest area (shown as rectangles in Fig. 2). Regressions were then counted as how many times the earlier interest areas were entered from the later interest areas in the sequential reading order, such as later parts of the same sentence, later sentences in a paragraph or later paragraphs in the text.
- Skipped words are those not fixated while reading. Word skipping is regarded as a consequence of higher-level cognitive processing^56^. In particular, word skipping rate is found to be strongly associated with word length and word predictability^57^. On the other hand, as suggested by an influential eye movement analysis model named Saccade-generation With Inhibition by Foveal Targets (SWIFT)^58,59^, fixation durations before skipped words are often inflated, which allows the skipped words being processed in the parafoveal visual area. This study adopted two measures related to word skipping: (1) Total Word Skipping Rate is at global passage-level; and (2) First Pass Dwell Time before Skipped Words is at local word-level.
- Pupillary response is an involuntary physiological reflex^52^ that can indicate arousal, stress, and mental workload. Enlarged pupil size was found to be related to increased cognitive load or arousal^60,61^. Pupil size may vary due to luminance and individual difference and thus a baseline pupil size is often measured for each participant. Following Zu et al.^62^, we included Change of Pupil Size (PS), defined as (PS_average_ − PS_baseline_)/PS_baseline_, for each participant on each passage in this study. In particular, PS_average_ is the average pupil size (in the unit of area) on words/phrases in a passage across all non-blink samples, while PS_baseline_ (in the unit of area) was collected during drift correction before reading a passage.
- Blinking is a common behavior that can be involuntary and voluntary^52^. It has been regarded as a physical embodiment of attentional decoupling process, in which the closure of eyelids can help shield internal thoughts from visual information^63^. Blink rate, the number of blinks per minute, is found to be associated with mental workload, fatigue, and state of attention^52^. A low blink rate can indicate a low level of tiredness or a high level of cognitive load, and thus was included in this study^52^.

Table 2 summarizes the aforementioned eye movement measures to capture participants’ cognitive processes and mental efforts. According to previous research^17,31,64^, four of these measures were *lexical ones in the early stage*, reflecting unconscious word recognition, lexical access, and automatic text processing. They are measures of the first pass eye movements at word-level, including First Fixation Duration, First Pass Dwell Time, First Pass Saccade Amplitude, and First Pass Dwell Time before Skipped Words. All these lexical measures were aggregated as mean value per passage, resulting in 9 values for each lexical measure per participant. Another four measures were *post-lexical ones in the late stage*, including Total Fixation Count, Total Fixation Duration, Total Regression Count, and Total Word Skipping Rate. They provide insights on conscious information integration and controlled processing. All these post-lexical measures were in the passage level, and thus there were 9 values for each post-lexical measure per participant. The remaining two variables, Change of Pupil Size and Blink Rate, are beyond eye movement but reflect readers’ cognitive processing as well^52,63^.

**Table 2:** Definition of eye-tracking measures.

Measures	Description	Implications on learning/cognition
Lexical processing stage		
First fixation duration	Length of time spent on first fixating on a word	These four measures are at local word-level. Longer fixation and dwell time and shorter saccade amplitude at this level can reflect increased cognitive load in unconscious word recognition, lexical access, and automatic text processing^17,31,64^
First pass dwell time	Aggregated duration of all first pass fixations on a word	
First pass saccade amplitude	Amplitude of the first saccade into a word	
First pass dwell time before skipped words	Summed durations of all first pass fixations on a word that is directly before skipped words	
Post-lexical processing stage		
Total fixation count	Total number of fixations on each passage	These four measures are at global passage-level. More fixations and regressions, longer fixation duration, and lower word skipping rate at this level can reflect increased cognitive load in conscious information integration and controlled processing^17,31,64^
Total fixation duration	Aggregated fixation durations of each passage	
Total regression count	Number of all inter-word regressions within a passage	
Total word skipping rate	Number of skipped words divided by total count of words in a passage	
Other		
Change of pupil size	Difference between average pupil size and pupil size baseline divided by pupil size baseline in a passage	Change of pupil size can indicate arousal, stress, and mental workload^15,52,60,61^
Blink rate	Number of blinks per minute	Blink rate can indicate mental workload, fatigue, and state of attention^52^

### Learner characteristics

The following characteristics of learner were examined in this study.

*Language Ability* was assessed by LexTale test^65^ which gauges participants’ familiarity level to English words and can reflect their general English proficiency^65^. The test includes 60 trials each presenting a string of letters. Participants were to determine whether the string in each trial was an existing English word or not. The LexTale score was computed as the proportion of correct responses in the test.

*Working Memory Capacity* was measured by the two-back test designed by Lau et al.^66^. Participants were required to keep track of a continuous stream of single letters appearing at different locations on the computer screen. They were asked to decide whether the current letter (in the verbal subtask) or the current location (in the spatial subtask) was the same as the one presented two trials before. Each subtask contained 36 trials, with the letter appearing for 1000 ms in each trial, followed by a 2500 ms blank screen. Working memory capacity was measured by the average accuracy of all two-back trials across verbal and spatial tests.

*Music Experience* was measured with the following two metrics.

- *Background Music Listening Habits*^15,67^ assessed the frequency of listening to BGM in daily self-learning/reading. The question was in a 5-point Likert scale: 1 = Never, 2 = Rarely, 3 = Sometimes, 4 = Often, 5 = Always.
- *Music Training Qualification*^67^ referred to whether participants have passed any music qualification exam (e.g., ABRSM, Trinity, National Conservatory of Music exams). These exams require systematic music training which may influence one’s music listening preferences and habits. Music qualification was coded into binary scale: No = 0, Yes = 1.

*Extraversion* as a salient personality dimension was measured with a subscale of the Ten-Item Personality Inventory (TIPI)^68^. Based on a mini-review, Küssner^8^ suggested that listening to BGM could have different impacts on cortical arousal of extraverts and introverts, and it could thus lead to differences in their learning outcomes. Developed from Big Five test^69^, TIPI is a time-economical survey with sufficient validity and reliability^40^. The extraversion subscale comprises a pair of traits (“extraverted & enthusiastic” versus “reserved & quiet”) on a seven-point Likert scale (1: strongly disagree; 7: strongly agree). Participants needed to rate to what extent the traits applied to them, and their ratings on the two traits were combined into one Extraversion score.

### Data preprocessing

Eye movements data preprocessing comprises the following stages: First, Eyelink 1000 plus’s automatic parser with default settings for cognitive research (c.f. Apparatus in Methods) was utilized to detect saccades and fixations^70^. Second, eye-tracking measures adopted in this study were then calculated using Eyelink DataViewer software. Third, outlying eye movements, such as those outside the stimuli (i.e., passage) image and those caused by drift correction, were removed. In preprocessing the WMC measure, the accuracies of the two 2-back subtests (i.e., verbal and spatial) were averaged. Two outliers were identified owing to a large discrepancy (over 30%) in accuracies of the two subtests. Cronbach’s Alpha for WMC test accuracy reaches 0.78 after outlier removal, indicating that internal consistency is acceptable. Last but not least, all measures of reading task performance, metacognition, eye movements were averaged across 9 passages, resulting in one value per participant for each measure in subsequent analysis.

### Data analysis

To benchmark the BGM and Silence groups, we used Mann-Whitney U tests (for ordinal variables such as BGM Listening Frequency and Extraversion) and independent sample T-tests (for numerical variables such as English ability and working memory capacity) to compare individual factors. This benchmarking can verify the BGM and Silence groups were comparable in individual factors.

To answer RQ1, differences on various measures between the conditions with and without BGM, we utilized independent sample T-tests or Mann-Whitney U tests to compare reading task performance, eye movement measures (numerical variables) and metacognition (ordinal variables) between the BGM and Silence groups. Benjamini-Hochberg procedure^71^ with a false discovery rate of 0.05 was used to control potential Type I error caused by multiple comparisons. Moreover, G * Power was adopted to perform Posterior Power analysis (α error probability = 0.05, sample size of each group = 50). In particular, since G * Power only enables calculating posterior power using Cohen’s d for Mann-Whitney tests, we converted correlation r to Cohen’s d using the online effect size converter at www.escal.site.

To answer RQ2, relationships between learners’ individual factors and various measures, multiple linear regression models were fit to predict reading task performance, metacognitive ratings, and eye movement measures (as dependent variables) using individual factors including English ability, working memory capacity, training on music, BGM listening frequency, and extraversion (as independent variables) in each background audio condition (with vs. without BGM). Simultaneous F tests with a significant level of 0.05 were used to correct multiple comparisons^72^.

All statistical analyses were carried out using JASP (jasp-stats.org). Both classical and Bayesian statistical analyses were adopted. In Bayes Factor analyses, parameters were configured using their default prior options in JASP: for t-tests, scaled information prior option with scale r = 0.707 was utilized given that a medium effect size was expected^73^; for linear regressions, Jeffreys-Zellner-Siow Cauchy prior option with scale r = 0.354 was applied^74^. According to Raftery’s recommendation, we reported BF (Bayes Factor) between 1 and 3 as weak, 3–10 as moderate (also known as positive), 10–100 as strong, and > 100 as very strong evidence for the hypothesis^75^.

Audio recordings of the interviews were transcribed and coded adopting thematic content analysis^76^. As only the BGM group listened to music during the experiment, we only analyzed their interview transcripts (N = 50) in this study. Specifically, from the transcripts we extracted excerpts which were defined as units of utterance with independent meanings. We then developed a coding framework concerning effects of BGM on learning with grounded theory approach^77^ through iterative examination of the transcripts. Moreover, quotations from interviews were utilized to supplement findings of quantitative research.

## Results

### Matching the two participant groups

A total of 102 participants were recruited in the study. Data collected from two of them were excluded from analysis due to device malfunction. The 100 remained participants (49 males, 51 females) came from diverse majors such as education, psychology, engineering, geography, pharmacy, mathematics, etc. Participants were allocated into a Silence group without music (25 males, 25 females) and a BGM group with preferred music while reading (24 males, 26 females). Mean age and standard deviation (SD, in parenthesis) in the Silence group was 22.2 (3.6) and 25.0 (4.2) in the BGM group. Twenty-three (23) participants in the BGM group and 19 in the Silence group had passed graded/qualification exams in music. The two groups were comparable (examined by two-tailed tests) in English ability as gauged by LexTale scores (t(98) = 0.419, *p* = 0.676, effect size d = 0.084) working memory capacity by N-back tasks (t(98) = 0.644, *p* = .521, d = 0.129); frequency of listening to background music during reading or self-learning, (U = 1172, *p* = 0.569, effect size r = − 0.063); and extraversion by the TIPI subscale (U = 1209, *p* = 0.773, r = 0.033). Therefore, the participants in the BGM and Silence groups were comparable in related individual factors.

### Analysis on music stimuli

Table 3 displays the music features analyzed from the playlists provided by students in the BGM group (N = 50). Genre and “Verbal vs. Instrumental” (whether having lyrics) were determined by manual coding and consensus protocol. In particular, genre was coded grounded on a genre classification taxonomy^78^. Two researchers first coded it independently, followed by a discussion. Items remained disagreed were then judged by a third researcher and coded by majority vote. Tempo (measured by beats per minute) was extracted from signals of digital music files employing Librosa^79^, a well-known audio processing library. The tempo values were further categorized as “slow, slow moderate, fast moderate, fast” based on the ranges given by extant literature^80^. As shown in Table 3, Rhythmic and Intensity was the most prevalent category which included the genres of pop, hip-hop (56%), followed by classical music (26%), while the remaining categories only accounted for smaller proportions. Most (60%) of the self-selected BGM were with lyrics while 32% was instrumental, with 8% containing both instrumental and verbal music. While fast (> 173 BPM) or slow (< 94 BPM) music was rarely used, fast moderate music (95 ~ 126 BPM) was more popular (54%) than slow moderate pieces (127 ~ 172 BPM) (28%).

**Table 3:** Music features on the tracks students selected.

Features	Definitions	Statistics
Genre category	The dominant genre category appearing most often in a BGM playlist.	Rhythmic and Intense category (e.g., genres of pop, hip-hop music; 56%; N = 28); Classical (24%; N = 12); Rebellious category (e.g., genres of heavy metal, punk, classic rock; 6%; N = 3); Easy listening (6%; N = 3); Electronic (2%; N = 1); Jazz and Blues (2%; N = 1); Others (4%; N = 2).
Verbal vs. Instrumental	Whether and to what extent lyrics exist in a BGM playlist.	Verbal (60%; N = 30); Instrumental (32%; N = 16); Playlist containing both verbal and instrumental (8%; N = 4).
Tempo	Number of beats per minute.	Fast moderate (54%; N = 27); Slow moderate (28%; N = 14); Fast (12%; N = 6); Slow (6%; N = 3).

### Reading task performance and metacognition

To answer RQ1, reading task performance and metacognition measures were compared with *two-tailed* t- and U-tests between the BGM and Silence groups. The means and SD (in parenthesis) as well as test results, effect sizes, BF_10_ are shown in Table 4.

**Table 4:** Results of T-tests on reading task performance and results of U-tests on metacognition.

Measures	BGM (experimental)	Silence (control)	t (98)	Effect size d	BF_10_
Reading task performance					
Passage Comprehension Accuracy	.474 (.150)	.429 (.149)	1.523	.305	.588
Passage Reading Time (s)	126 (51.6)	106 (47.2)	2.082*	.416	1.416

Measures	BGM (experimental)	Silence (control)	U (98)	Effect size r	BF_10_
Metacognition					
Engagement	3.26 (.78)	3.13 (.80)	1125	.100	.263
Difficulty	3.20 (.54)	3.27 (.63)	1345	− .076	.240
Understanding	2.90 (.57)	2.87 (.67)	1260	− .008	.195

N = 100 (BGM Group = 50; Silence Group = 50). *p values < 0.05 were at significant level. Effect size is given by Cohen’s d (for t-test) and rank biserial correlation r (for Mann-Whitney u-test).

There was no significant difference on passage comprehension accuracy between the two groups (t(98) = 1.523, *p* = .131, *d* = .305, observed power = 0.327; BF_01_ = 1.700, indicating weak evidence favoring the null hypothesis). The BGM group spent significantly longer time (in the unit of second) on reading the passages than the Silence group (t(98) = 2.082, *p* = .040, d = .416, observed power = 0.540; BF_10_ = 1.416, indicating weak evidence favoring the alternative hypothesis). This indicated that longer time may be required to achieve similar level of comprehension if students listened to their self-selected BGM.

Regarding metacognitive measures, no significant differences were found between the two groups in engagement (U = 1125, *p* = .388, r = .100, observed power = 0.169; BF_01_ = 3.802, indicating moderate evidence favoring the null hypothesis), difficulty (U = 1345, *p* = .511, *r* = − .076, observed power = 0.117; BF_01_ = 4.167, indicating moderate evidence favoring the null hypothesis), understanding (U = 1260, *p* = .948, r = − .008, observed power=0.051; BF_01_ = 5.128, indicating moderate evidence favoring the null hypothesis).

### Reading process: eye-tracking data

The results of comparing eye-tracking measures with one-tailed t-tests between the groups are shown in Table 5.

**Table 5:** Results of T-tests on eye movement behaviors.

Measures	BGM (experimental)	Silence (control)	t (98)	Effect size d	BF_+0_	BF_-0_
Lexical						
1st fixation duration (ms)	227 (33)	225 (25)	.286	.057	0.265	–
1st pass dwell time (ms)	279 (47)	274 (39)	.593	.119	0.351	–
1st pass saccade amplitude (°)	5.3 (.84)	5.5 (.98)	− 1.031	− .206	–	0.563
1st dwell time before skip (ms)	282 (51)	277 (44)	.518	.104	0.326	–
Post-lexical						
Total fixation count	457 (185)	381 (162)	2.199*	.440	3.447	–
Total fixation duration (ms)	101,355 (42,142)	83,779 (38,001)	2.190*	.438	3.390	–
Total regression count	105.1 (49.9)	87.8 (43.1)	1.854*	.371	1.840	–
Total skip rate (%)	.257 (.097)	.300 (.109)	− 2.064*	− .413	–	2.669
Other						
Change of pupil size (%)	.109 (.134)	.120 (.116)	− .425	− .085	0.158	–
Blink rate (per minute)	21.8 (13.9)	23.1 (12.4)	− .512	− .102	–	0.324

N = 100 (BGM Group = 50; Silence Group = 50). *p values < 0.05 were at significant level.

As for lexical measures, t-tests revealed no significant difference (at p < 0.05 level) between the groups, with BF analyses providing weak to moderate evidence for the null hypothesis (BF_0+_ or BF_0-_ values ranging from 1.776 to 3.774). In other words, BGM had little impact on lexical processing in the first pass. When it comes to post-lexical measures, we observed significant differences between the two groups. The BGM group overall had more fixations (t(98) = 2.199, *p* = .015, *d* = .440, observed power = 0.705; BF_+0_ = 3.447, indicating moderate evidence favoring the alternative hypothesis), longer fixation time (t(98) = 2.190, *p* = .015, d = .438, observed power = 0.702; BF_+0_ = 3.390, indicating moderate evidence favoring the alternative hypothesis), and higher regression count (t(98) = 1.854, *p* = .033, d = .371, observed power = 0.578; BF_+0_ = 1.840, indicating weak evidence favoring the alternative hypothesis) than the Silence group. Moreover, the BGM group had significantly lower skipping rate than the Silence group (t(98) = − 2.064, *p* = .021, d = − .413, observed power = 0.658; BF_−0_ = 2.669, indicating weak evidence favoring the alternative hypothesis). The two groups did not differ on average variation of pupil size during reading or blink rate based on either classical t-tests or Bayesian factor analysis.

### Predicting reading task performance, metacognition and eye movement from individual traits

To answer RQ2, we applied multiple linear regression models to estimate which learner characteristics could predict reading task performance, metacognition, and eye-movement metrics. Tests for multicollinearity revealed that the predictors had a very low level of multicollinearity (all VIF < 2). For classical regression analyses, each predictor’s standardized coefficients β, and each regression model’s effect size R^2^ are shown in Tables 6 and 7 for BGM and Silence groups respectively. For Bayesian analyses, each predictor’s BF_inclusion_ and each regression model’s BF_10_ are shown in Tables 8 and 9 for BGM and Silence groups respectively.

**Table 6:** Classical linear regression models of BGM group (Experimental).

Reading task performance	β					R^2^
	ESL proficiency	BGM Freq	WMC	Music train	Extraversion	
Passage Comprehension Accuracy	.071	− .201	.072	.195	.095	.089
Passage Reading Time	− .415**	− .323*	.270*	.080	− .069	.339**

Metacognition	β					R^2^
	ESL proficiency	BGM Freq	WMC	Music train	Extraversion	
Engagement	.066	− .215	.117	− .075	.173	.084
Difficulty	.235	− .150	.294	.069	.213	.178
Understanding	.155	.122	− .045	− .189	− .271	.140

Eye movement	β					R^2^
	ESL proficiency	BGM Freq	WMC	Music train	Extraversion	
Lexical						
1st fixation duration	− .205	− .006	− .107	− .115	.123	.106
1st pass dwell time	− .458**	− .008	− .112	− .132	.111	.311**
1st pass saccade amplitude	.480**	.056	.183	− .005	− .219	.329**
1st dwell time before Skip	− .456**	.019	− .080	− .151	.074	.305**
Post-lexical						
Total fixation count	− .400*	− .309*	.278*	.088	− .098	.321**
Total fixation duration	− .378*	− .309*	.252	.024	− .002	.315**
Total regression count	− .252	− .293*	.315*	.138	− .195	.264*
Total skip rate	.344	.249	− .165	− .050	− .047	.215
Other						
Change of pupil size	.170	.058	.072	− .059	− .113	.047
Blink rate	− .349	− .139	.127	.288	− .312	.197

*p < .05; **p < .01. *Note.* *ESL Proficiency:* ESL learners’ English proficiency measured by LexTale test, *BGM Freq.:* BGM listening frequency in daily life, *WMC:* Working memory capacity measured by N-back task, *Music Train:* Training on music, *Extraversion:* Extraversion measured by TIPI subscale.

**Table 7:** Classical linear regression models of silence group (Control).

Reading task performance	β					R^2^
	ESL proficiency	BGM Freq	WMC	Music train	Extraversion	
Passage Comprehension Accuracy	.089	− .109	.240	.018	− .113	.112
Passage Reading Time	− .318	.178	.152	.079	− .132	.161

Metacognition	β					R^2^
	ESL proficiency	BGM Freq	WMC	Music train	Extraversion	
Engagement	− .061	− .179	.234	− .072	− .020	.072
Difficulty	.260	− .019	.242	− .016	.055	.144
Understanding	− .289	− .091	.019	− .123	− .141	.109

Eye movement	β					R^2^
	ESL proficiency	BGM Freq	WMC	Music train	Extraversion	
Lexical						
1st fixation duration	− .256	− .184	.062	.046	.144	.092
1st pass dwell time	− .457**	− .085	.047	− .003	.105	.212*
1st pass saccade amplitude	.598***	.076	− .129	.070	.056	.339**
1st dwell time before skip	− .445**	.051	.030	.059	.067	.216*
Post-lexical						
Total fixation count	− .284	.193	.132	.032	− .139	.148
Total fixation duration	− .322	.163	.122	.049	− .096	.149
Total regression count	− .161	.262	.097	.077	− .181	.122
Total skip rate	.315	− .026	− .195	.015	.030	.122
Other						
Change of pupil size	.185	.108	− .136	.075	− .083	.055
Blink rate	− .092	.117	.186	.186	− .155	.097

Please refer to Note below Table 6 for abbreviation definition. *p < .05; **p < .01; ***p < 0.001.

**Table 8:** Bayesian linear regression models of BGM group (Experimental).

Reading task performance	BF_inclusion_					BF_10_
	ESL proficiency	BGM Freq	WMC	Music train	Extraversion	
Passage comprehension accuracy	0.241	0.312	0.231	0.345	0.227	0.070
Passage reading time	10.501	5.698	3.763	0.986	0.961	14.575

Metacognition	BF_inclusion_					BF_10_
	ESL proficiency	BGM Freq	WMC	Music train	Extraversion	
Engagement	0.208	0.350	0.246	0.128	0.265	0.065
Difficulty	0.618	0.491	1.066	0.468	0.562	0.360
Understanding	0.383	0.328	0.325	0.383	0.897	0.175

Eye movement	BF_inclusion_					BF_10_
	ESL proficiency	BGM Freq	WMC	Music train	Extraversion	
Lexical						
1st fixation duration	0.534	0.246	0.271	0.343	0.323	0.094
1st pass dwell time	38.624	0.353	0.451	0.478	0.420	6.973
1st pass saccade amplitude	33.725	0.476	0.791	0.469	0.957	11.161
1st dwell time before skip	37.324	0.338	0.387	0.499	0.364	6.037
Post-lexical						
Total fixation count	6.587	4.305	3.736	0.981	1.007	8.987
Total fixation duration	7.944	3.918	2.609	0.816	0.797	7.713
Total regression count	1.099	2.223	3.315	0.783	0.001	2.254
Total skip rate	2.369	1.036	0.787	0.509	0.520	0.771
Other						
Change of pupil size	0.234	0.170	0.172	0.170	0.214	0.035
Blink rate	0.859	0.573	0.609	0.758	0.940	0.527

Please refer to Note below Table 6 for abbreviation definition.

**Table 9:** Bayesian linear regression models of silence group (Control).

Reading task performance	BF_inclusion_					BF_10_
	ESL proficiency	BGM freq	WMC	Music train	Extraversion	
Passage comprehension accuracy	0.333	0.315	0.556	0.266	0.339	0.106
Passage reading time	1.052	0.530	0.466	0.356	0.398	0.258

Metacognition	BF_inclusion_					BF_10_
	ESL proficiency	BGM Freq	WMC	Music train	Extraversion	
Engagement	0.192	0.239	0.330	0.196	0.197	0.052
Difficulty	0.838	0.316	0.750	0.312	0.316	0.189
Understanding	0.588	0.257	0.245	0.294	0.303	0.099

Eye movement	BF_inclusion_					BF_10_
	ESL proficiency	BGM Freq	WMC	Music train	Extraversion	
Lexical						
1st fixation duration	0.391	0.261	0.216	0.221	0.265	0.073
1st pass dwell time	8.272	0.358	0.343	0.338	0.390	0.731
1st pass saccade amplitude	204.394	0.320	0.407	0.321	0.331	14.603
1st dwell time before skip	9.427	0.352	0.336	0.343	0.362	0.791
Post-lexical						
Total fixation count	0.808	0.543	0.397	0.321	0.369	0.201
Total fixation duration	1.109	0.477	0.387	0.321	0.340	0.207
Total regression count	0.353	0.562	0.301	0.217	0.343	0.124
Total skip rate	0.783	0.293	0.402	0.277	0.272	0.126
Other						
Change of pupil size	0.240	0.176	0.194	0.185	0.185	0.040
Blink rate	0.231	0.245	0.349	0.299	0.282	0.081

Please refer to Note below Table 6 for abbreviation definition.

The regression model comprising learner characteristics only weakly explained the variation in passage comprehension accuracy in both BGM (*R*^2^ = 0.089, F(5, 43) = 0.838, *p* = 0.530) and Silence groups (*R*^2^ = 0.112, F(5, 43) = 1.090, p = 0.380). The evidence better supported the null model (BF_10_ = 1) as compared to alternative model in BGM (BF_10_ = 0.070) and Silence group (BF_10_ = 0.106). Furthermore, in neither BGM group nor Silence group did we observe any evidence supporting the relationships between learner characteristics and passage comprehension accuracy (all BF_inclusion_ < 1).

In the BGM group, passage reading time and several eye movement measures were significantly correlated with learner characteristics (Table 6) and there was evidence from Bayesian factors in favor of these relationships (Table 8), while fewer such correlations (Table 7) were observed in the Silence group and fewer Bayesian evidence (Table 9) favoring these relations. For example, in the BGM group there were negative correlations between General English Proficiency (measured by LexTale test scores) and Reading Time (β = − .415, *p* = .007; BF_inclusion_ = 10.501), Total Fixation Count (β = − .400, *p* = .010; BF_inclusion_ = 6.587), and Total Fixation Duration (β = − .378, *p* = .015; BF_inclusion_ = 7.944), as well as negative correlations between Frequency of BGM use during self-learning/reading and Reading Time (β = − .323, *p* = .015; BF_inclusion_ = 5.698), Total Fixation Count (β = – .309, *p* = .022; BF_inclusion_ = 4.305), Total Fixation Duration (β = − .309, *p* = .022; BF_inclusion_ = 3.918), and Total Regression Count (β = − .293, *p* = .036; BF_inclusion_ = 2.223). Also significant in the BGM group were the positive correlations between Working Memory Capacity (measured by N-back task) and Reading Time (β = .270, *p* = .040; BF_inclusion_ = 3.763), Total Fixation Count (β = .278, *p* = .022; BF_inclusion_ = 3.736), and Total Regression Count (β =.315, *p* = .024; BF_inclusion_ = 3.315). None of these correlations (all *p* > 0.05) and Bayesian evidence supporting these relationships (all BF_inclusion_ < 1) were observed in the Silence group.

Some other correlations were identified in both BGM and Silence conditions, that is, General English Proficiency (measured by LexTale test scores) had positive relationships with three lexical-level eye movement measures: First Pass Dwell Time (BGM Group: β = − .458, *p* = .004, BF_inclusion_ = 38.624; Silence Group: β = − .457, *p* = .003, BF_inclusion_ = 8.272), First Pass Saccade Amplitude (BGM Group: *β* = .480, *p* = .002, BF_inclusion_ = 33.725; Silence Group: β = .598, *p* < .001, BF_inclusion_ = 204.394) and First Pass Dwell Time before Skipped Words (BGM Group: β = − .456, *p* = .004, BF_inclusion_ = 37.324; Silence Group: β = − .445, *p* = .004; BF_inclusion_ = 9.427). Bayesian factors showed *moderate to very strong* evidence supporting these relationships.

However, in neither BGM nor Silence conditions did we observe significant correlations between another two tested learner characteristics (Training on Music, Extraversion) and measures of reading task performance, metacognition, or eye movements based on both classical hypothesis testing (Tables 6, 7) and Bayesian factor approaches (Tables 8, 9).

### Interview responses

Two primary categories emerged from the thematic analysis: effects of BGM on cognition and effects of BGM on emotion. A total of 280 excerpts were extracted from the responses and 29% (82 excerpts) were double coded by two independent researchers who were cognitive science graduate students. The inter-rater reliability was 0.94, as measured by Cohen’s kappa coefficient, implying an excellent level of agreement between coders^81^. The resultant codes with definitions, participant counts, and excerpt counts are presented in Table 10. There are 152 excerpts about the effects of BGM on cognition and 128 on emotion.

**Table 10:** Thematic analysis results of interviews.

Code	Definition	Count of participants	Count of excerpts
Category I: effects of BGM on cognition		**50**	**152**
Sub-category: benefits		*21*	*42*
Improve concentration	Participant mentioned BGM helped improve concentration	18	24
Block out noise	Participant mentioned BGM helped them block out noise in the environment	12	16
Improve efficiency	Participant mentioned BGM helped improve efficiency	2	2
Sub-category: harm		*41*	*87*
Distract concentration	Participant mentioned BGM distracted them from reading	41	87
Sub-category: neutral		*6*	*7*
Read for longer	Participant mentioned they read for a longer period of time when there was BGM	6	7
Sub-category: no impact		*13*	*16*
No effect on info processing	Participant mentioned listening to BGM did not affect information processing	13	16
Category II: effects of BGM on emotion		**50**	**128**
Sub-category: benefits		*40*	*108*
Increase happiness	Participant mentioned BGM increased their happiness	19	27
Arouse energy	Participant mentioned BMG aroused their energy	15	26
Relax	Participant mentioned feeling relaxed or comfortable from listening to BGM	17	23
Calm down	Participant mentioned BGM calmed them down	12	17
Feel enjoyment	Participant mentioned they derived enjoyment from BGM	9	10
Increase engagement	Participant mentioned their engagement increased from listening to BGM	4	5
Sub-category: harm		*9*	*13*
Being annoyed	Participant mentioned annoyed or uncomfortable from listening to BGM	6	8
Tiredness and sleepiness	Participant mentioned tired and sleepy feeling from listening to BGM	3	5
Sub-category: neutral		*6*	*7*
No effect on mood	Participant mentioned listening to BGM did not affect mood	6	7

Aggregated values for each Category and Sub-category are in bold and italics respectively.

Some participants (42%) mentioned cognitive benefits of listening to BGM, such as music “helped me concentrate on what I was reading” (Participant #14), “blocked out noise in the environment” (#32), and “facilitated me to have a more efficient study” (#26). However, 41 (82%) of the participants reported negative effects of BGM on cognition, such as “music distracted my attention” and “I was unable to devote myself completely to the reading task” (#58). In particular, distraction might occur when the tracks contained lyrics, just as one participant commented: “I can't really read the words because I’ll pay attention to the lyrics.” (#53). Some impacts were neutral such as “music listening increased reading time” (#39) and “I would like to read longer as my favourite BGM was played” (#47). In addition, no impact was also mentioned. Several participants stated BGM had no impact on information processing, because “I was unaware that music was playing while I was reading” (#26), or they were able to “multitask by listening to music and reading at the same time” (#12).

The participants indicated benefits of listening to BGM on emotion, such as “[listening to my favourite songs] increased my happiness” (#55), “aroused my energy” (#94), “I felt more relaxed and more comfortable” (#54), and “music calmed me down and helped me get into reading states” (#100). Nonetheless, several participants reported feeling “annoyed or uncomfortable” by “fast-tempo music” (#71) or “rock music” (#57), whereas some commented that “instrumental classical music” or “calming music” made them feel "tired and sleepy" (#94, #101). Last but not least, nearly one tenth of respondents stated that BGM had no effect on their mood (#73).

## Discussions

### Effects of BGM on reading task performance and metacognition

First, there were no significant differences in passage comprehension accuracy, or metacognition measures between the BGM and Silence groups (Table 4). The results on passage comprehension accuracy were consistent with prior research^15,16^, with Bayesian factor analysis providing weak evidence for the null hypothesis in passage comprehension accuracy between the two groups. As few existing studies examined participants’ metacognition during reading, the finding that listening to self-selected BGM did not affect self-perceived metacognition fills a research gap.

Compared to the Silence condition, participants in the BGM condition spent significantly longer time reading the passages (Table 4). It can be speculated that BGM may increase cognitive burdens of participants, and thus more reading time is needed to compensate for the increased workload while achieving similar level of comprehension^16,25,82^. It may also be due to known functions of music that affects temporal perception^83^, meaning that listeners tend to alter their sense of time^84^. Through the thematic analysis of interview data (Table 10), we found that listening to music yielded hedonic values (e.g., experiencing pleasure) and helped learners maintain positive emotions such as happiness, aroused energy, relaxation, comfort, increased engagement in reading^4,67,83^, which might have helped participants endure longer reading time.

### Effects of BGM on eye movements

Reading with or without BGM overall demonstrated quite similar patterns in first pass measures which were related to lexical access and world-level processing (Table 5), suggesting that certain oculomotor control mechanisms relevant to word recognition remained functional despite music exposure^15,16^, probably because reading behavior has become automated actions after extensive exposure to texts throughout years of education^34^. This is in accordance with the results in Johansson et al.’s research^15^ which found no main effect on first pass fixation duration or first pass saccade amplitude among distinct background audio conditions (e.g., preferred/non-preferred music, noise and silence). In this regard, listening to self-selected BGM did not seem to increase learners’ difficulties at lexical level.

Compared to the Silence group, reading with BGM involved a larger fixation count, a larger regression count, longer fixation durations, and fewer word skipping behaviors (Table 5) which were typical eye movement patterns of worse post-lexical information processing (e.g., integration from word recognition to text comprehension)^17^. As eye-tracking measures reflect cognitive load during the reading process^13,32^, the results indicate that participants in the BGM group would experience a higher level of extraneous cognitive load than those in the Silence group. Specifically, longer fixation durations, more fixations, and more regressions indicate high cognitive load in current reading task^15,53^. Previous research has pointed out that extensive rereading behaviors as reflected by a larger regression count might serve to compensate for the music disruption^16^. According to the E-Z Reader model^20^, most regressions are due to difficulties in post-lexical processing. In these regards, our results support that BGM affects oculomotor behaviors during reading comprehension by increasing the difficulty of post-lexical/semantic integration, which is consistent with previous research findings that BGM can make semantic integration more challenging by evaluating the effect of BGM on neural responses^6^. Through separating fine-grained eye movement measures into lexical ones in the early stage and post-lexical ones in the late stage, this study discovered how BGM affected different stages of reading comprehension, namely BGM influenced semantic integration rather than lexical access.

Moreover, we found that learners in the BGM condition skipped fewer words than those in silence condition, which differs from a previous study by Zhang et al.^16^ where participants’ word skipping behaviors were found to be the same in BGM and Silence conditions. This discrepancy might possibly be related to participants’ language proficiency: Zhang et al.’s study^16^ were native English speakers while those in this study were second language learners. Previous study has revealed that first-language readers skipped more words than non-native readers in silence^85^, but there are few studies further comparing native and non-native readers’ word skipping behaviors in the BGM condition. It could be speculated that, due to higher language proficiency and familiarity, first-language readers’ skipping rate could be less affected by BGM compared to non-native speakers. While this speculation could be partially supported by the positive correlation between LexTale scores and skipping rate in the BGM condition, Pearson r(50) = .364, *p* = .009, further studies are needed to compare the effects of BGM on first- and second- language learners.

Last but not least, there were no between-group differences in change of pupil size and blink rate. These involuntary oculomotor behaviors are related to not only cognitive load, but also arousal, stress and fatigue^15,52^. These effects might be cancelled out by one another and thus resulted in no between-group difference. More studies are needed to investigate this speculation by measuring these constructs separately.

Overall, even though results indicated that BGM had little influence on passage comprehension accuracy, eye movement measures revealed that students in the BGM group might have experienced heavier cognitive load in post-lexical semantic integration than those in the Silence group. Considered as a whole, the present results, at least partially, supported the assumption regarding extraneous cognitive load in Cognitive Load Theory^21^: Certain reading process could be impeded by extraneous cognitive load imposed by learners’ self-selected BGM. This effect of BGM on reading comprehension can be explained by its tendency to cause distraction when BGM is not strategically integrated to the reading task^26^. 82% participants in the BGM group reported being distracted by BGM in the interview, even though BGM brought emotional benefits to them (e.g., increased happiness and enjoyment). However, there was no quantitative analysis of whether learners enjoyed the music more than the reading task, which can be investigated in future work. In addition, our interviews also revealed that certain types of BGM might be detrimental to reading comprehension. For example, fast-paced or rock music might be overstimulating learners when they were reading; verbal music (i.e., music with lyrics) might have produced some cognitive dissonance at least for some learners. Future research is needed to verify or investigate these possible mechanisms of BGM.

On the other hand, evidence from eye-tracking furthers our understanding on how BGM may affect the reading process: It influences post-lexical integration (i.e., more controlled processing at the semantic and global level) rather than lexical access (i.e., involuntary, and more automatic processing at the word level)^16^. This finding on different stages of the reading process can only be teased out by fine-grained analysis on eye movement measures. It furthers our understanding of *how* BGM affects reading comprehension, which can help future research on human behavior, perception, and cognition in complex learning contexts.

### Learners with different english proficiency levels

In the BGM condition, students with higher English proficiency spent less reading time, less first pass dwell time (a lexical measure), fewer fixation counts, and shorter fixation durations (post-lexical measures) than those with lower English proficiency (Table 6), whereas such relationships were not observed in the silence condition (Table 7). The effect sizes of these relationships (β values) were between − 0.458 and − 0.378 (Table 6), suggesting that, in the BGM condition, a one standard deviation increase in a student’s English proficiency was associated with an around 0.4 standard deviation decrease in reading time, first pass dwell time, fixation counts, and fixation durations; Bayesian Factor analyses provided moderate to strong evidence in favor of these relationships with BF_inclusion_ values ranging from 6.587 to 10.501 (Table 8). Given that these measures can indicate cognitive load in lexical and post-lexical processes^53^, it can be extrapolated that participants with greater English abilities have experienced less cognitive load in both word recognition and semantic integration than those with weaker English abilities when they read with BGM. The results match the conclusion of Williams and Morris’s research^35^ that indicates word familiarity affects eye movement behaviors during reading. It is noted that, although previous studies^86,87^ have tested language ability as a control variable and ensured that it would not confound the interpretation of BGM’s effect, there has been little evidence on how language ability plays a role in reading with BGM. This paper fills the gap with eye-tracking evidence.

### Learners with different BGM listening frequency

Frequency of listening to BGM in daily self-learning/reading showed negative correlations with reading time, fixation count, regression count, and fixation durations in the presence of BGM (Table 6). The effect sizes of these relationships (β values) were between − 0.323 and − 0.293 (Table 6), indicating that those who were accustomed to listening to BGM could complete the reading task with fewer mental efforts when reading with BGM. Bayesian Factor analysis provided weak to moderate evidence for these relationships with BF_inclusion_ values between 2.223 and 5.698 (Table 8). This finding complements those of an earlier study that students who often played BGM while studying performed equally well on text comprehension in both BGM and silence conditions while those who were not used to BGM while reading performed better without BGM^42^. In the meantime, we also found that the habit of listening to BGM while learning was not related to any of the dependent variables in the Silence condition (Table 7), further confirming that having such habits would not affect task performance, metacognition or eye movements in reading without BGM^15^.

### Learners with different working memory capacity

No significant correlation between working memory capacity (WMC) and passage comprehension accuracy was observed in either BGM or Silence conditions. However, we found positive correlations between WMC and reading time, fixation count, and regression count in the BGM condition (Table 6) but not in the Silence condition (Table 7). The effect sizes of these relationships (β values) were between 0.270 and 0.315 (Table 6), suggesting that in the BGM condition a one standard deviation increase in a student’s WMC was associated with an around 0.3 standard deviation increase in reading time, fixation count, and regression count. Bayesian Factor analysis provided moderate evidence supporting these relationships with BF_inclusion_ values ranging from 3.315 to 3.763 (Table 8). As suggested by Atkinson-Shiffrin’s model^88^, WMC (also known as short-term memory storage) has a duration of up to 30 seconds, while in our study the average reading time for each passage was much longer than 30 seconds (Table 4). It could thus be inferred that the reading time of students in both BGM and Silence groups might not be entirely dependent on WMC. It also inspired us to refer to eye movement variables, as finer-grained temporal information^13^, to explain the positive relation between WMC and reading time in BGM but not Silence condition. Considering the fact that increased fixations and regressions are connected to in-depth text processing^32,33^, it can be inferred that students with higher WMC may have devoted more mental efforts to the texts than those with lower WMC when reading with their preferred BGM. Besides, it has been found that students with high WMC have the capacity to process both the reading task and the background auditory information^5^ and thus may need to look back more to compensate for incomplete processing^89^ caused by music distraction; whereas those with lower WMC may not be able to process both channels and thus chose to focus on reading only. This could be corroborated by the exit-interviews where some high-WMC participants in the BGM group commented that they “enjoyed the BGM” (Participant #84, 98, 102) and “intentionally revisited the places that were interrupted by BGM” (Participant #98), while some low-WMC participants acknowledged that they tried to “block out the music” (Participant #69, 92) so as to “concentrate on the text” (Participant #69, 73, 92). To the best of our knowledge, only one existing study utilized eye-tracking to investigate the role of WMC in the process of reading with BGM^15^. That study by Johanssan^15^ was on first-language learners and reported no significant correlation between WMC and either reading time or eye fixation/regression variables in any background audio conditions considered. Our findings add to the literature by examining second language learners who may need to draw more cognitive resources when reading with BGM^13,90^.

In summary, we found that, when reading with their self-selected BGM, learners with higher English proficiency, more frequent BGM usage during reading/self-learning, or lower WMC demonstrated eye movement patterns indicating a lower level of mental efforts. These findings can provide practical guidance for students to make personalized decisions on reading with BGM. First, students with high language proficiency and habit of listening to BGM in daily self-learning/reading would face little extra cognitive load, while enjoy benefits of BGM on longer enduring time. Second, students who are used to listening to BGM while studying can feel free to choose to study without BGM, since reading in silence will not affect their task performance, metacognition, or eye movements. Third, students with high WMC might face higher cognitive load when they read with BGM, but that does not affect their reading task performance or enjoyment with BGM.

Our findings have practical implications for teachers and instructional designers. First, learning environment designers can leverage students’ preferred music to increase their enjoyment and motivation in reading activities. For example, a learning management system (e.g., Moodle) or an e-reader application (e.g., Perusall) can play a student’s preferred study music when he/she opens a document to read, creating an enjoyable and positive learning atmosphere. However, the use of BGM during learning should consider the type of learning tasks and the characteristics of the BGM to reduce potential cognitive overload and dissonance. For instance, the use of instrumental music (without lyrics) in the reading task may add less cognitive load as compared to utilizing verbal music (with lyrics)^5^. Second, as listening to BGM may lengthen the time consumed for completing the reading task, BGM is not recommended when students have pressing deadlines. Third, the effects of self-selected BGM may vary across different types of learners (e.g., learners with high vs. low WMC), and therefore teachers or learning environment designers are advised to take learner characteristics into account when integrating music into the learning environment. For example, for students with low language proficiency and without habit of listening to BGM in daily self-study, teachers may suggest them limiting their usage of BGM while reading, as they would face higher cognitive load.

## Limitations and future directions

Participants of this study were English as second language learners in Hong Kong and thus the results may not be applicable to native English speakers or students in another region of the world. Future work may examine whether the current findings can be found among native English speakers and whether the effect of BGM is language-specific. This study is limited to the reading task in which students have autonomy to self-regulate their learning processes (i.e., they had unlimited time to read) with their self-selected music. It does not include music composed for specific learning tasks or strategically chosen by teachers. Our findings may not be generalized to other types of tasks or assessment episodes (e.g., viewing instructional videos, writing essays, solving math puzzles). Moreover, the reading materials were limited to passages in standard tests. While standard tests can be more accurate in measuring passage comprehension accuracy, reading materials in the reality are diverse including novels, poems etc, which can be considered in future studies. Besides, during the experiment, the researchers were aware of the conditions to which the participants were assigned to. Future work will improve the experiment design to maintain strict blinding. Last but not least, we may not have sufficient power to test for group difference on some variables (e.g., observed power for passage comprehension accuracy is 0.327). Future studies can examine the research questions with a larger sample size to better reveal the effect of music on reading (e.g., a sample size of 340 would be needed to find a statistically significant difference for two evenly divided groups in an independent sample t-test, with the observed effect size d = .305, 0.05 given alpha, 0.8 given power, and 2 tails).

## Conclusions

To examine the effects of self-selected BGM on reading comprehension, this study employed an eye-tracking approach to capture the learners’ reading processes, in both lexical and post-lexical levels. Through examining personal characteristics, this study also investigated individual factors related to the effects of BGM on reading comprehension. Results demonstrated some interesting findings. First, students in the BGM and Silence conditions showed no significant difference in passage comprehension accuracy or metacognition. Second, eye movement analysis revealed that listening to BGM imposed heavier cognitive load on post-lexical processes, but not on lexical processes. Third, in the presence of BGM, students with higher English proficiency, more frequent BGM usage, and lower WMC were found to have experienced less cognitive load.

## Data Availability

The datasets used and/or analysed during the current study available from the corresponding author on reasonable request.
